# Urinary Extracellular Vesicle Protein Profiling and Endogenous Lithium Clearance Support Excessive Renal Sodium Wasting and Water Reabsorption in Thiazide-Induced Hyponatremia

**DOI:** 10.1016/j.ekir.2018.09.011

**Published:** 2018-09-22

**Authors:** Sarath K. Channavajjhala, Roger Bramley, Theresa Peltz, Wilna Oosthuyzen, Wenjing Jia, Sue Kinnear, Barry Sampson, Nick Martin, Ian P. Hall, Matthew A. Bailey, James W. Dear, Mark Glover

**Affiliations:** 1 Division of Therapeutics and Molecular Medicine, University of Nottingham, Nottingham, UK; 2 NIHR-Nottingham Biomedical Research Centre, Nottinghamshire, UK; 3 Trace Element Laboratory, Imperial College Healthcare NHS Trust, Charing Cross Hospital, London, UK; 4 BHF Centre for Cardiovascular Science, University of Edinburgh, Edinburgh, UK

**Keywords:** diuretic, hypertension, hyponatremia, sodium, thiazide, urinary extracellular vesicles

## Abstract

**Introduction:**

Thiazide diuretics are among the most widely used antihypertensive medications worldwide. Thiazide-induced hyponatremia (TIH) is 1 of their most clinically significant adverse effects. *A priori* TIH must result from excessive saliuresis and/or water reabsorption. We hypothesized that pathways regulating the thiazide-sensitive sodium-chloride cotransporter NCC and the water channel aquaporin-2 (AQP_2_) may be involved. Our aim was to assess whether patients with TIH would show evidence of altered NCC and AQP_2_ expression in urinary extracellular vesicles (UEVs), and also whether abnormalities of renal sodium reabsorption would be evident using endogenous lithium clearance (ELC).

**Methods:**

Blood and urine samples were donated by patients admitted to hospital with acute symptomatic TIH, after recovery to normonatremia, and also from normonatremic controls on and off thiazides. Urinary extracellular vesicles were isolated and target proteins evaluated by western blotting and by nanoparticle tracking analysis. Endogenous lithium clearance was assessed by inductively coupled plasma mass spectrometry.

**Results:**

Analysis of UEVs by western blotting showed that patients with acute TIH displayed reduced total NCC and increased phospho-NCC and AQP_2_ relative to appropriate control groups; smaller differences in NCC and AQP_2_ expression persisted after recovery from TIH. These findings were confirmed by nanoparticle tracking analysis. Renal ELC was lower in acute TIH compared to that in controls and convalescent case patients.

**Conclusion:**

Reduced NCC expression and increased AQP_2_ expression would be expected to result in saliuresis and water reabsorption in TIH patients. This study raises the possibility that UEV analysis may be of diagnostic utility in less clear-cut cases of thiazide-associated hyponatremia, and may help to identify patients at risk for TIH before thiazide initiation.

Thiazide diuretics have been used in the management of hypertension for more than half a century.^1^ They lower blood pressure by inhibition of the sodium-chloride cotransporter (NCC) in the distal convoluted tubule and have all-cause mortality benefits equivalent to those of angiotensin-converting enzyme inhibitors and calcium channel antagonists.^1^, ^2^ Although thiazides are generally well tolerated, their use is limited in a minority of patients due to hyponatremia.^3^ Owing to the very large number of patients prescribed thiazides, thiazide-induced hyponatremia (TIH) is the most common cause of drug-induced hyponatremia requiring hospitalization.^4^, ^5^

The mechanisms underlying TIH remain unclear. Risk factors for TIH include advanced age, reduced body mass, and concurrent use of other medications that impair water excretion.^6^ *A priori* TIH must result from excessive saliuresis and/or water reabsorption (or ingestion). It therefore seems likely that excessive saliuresis and/or water reabsorption (or ingestion) occurs via altered regulation of NCC and/or aquaporin-2 (AQP_2_). As thiazide diuretics have not, to the best of our knowledge, been reported to produce saliuresis with a urinary sodium concentration greater than that of plasma, this suggests that excessive water reabsorption (or ingestion) is a fundamental part of the pathogenesis of TIH. Our aim in this study was to probe the molecular mechanisms underlying TIH by investigating whether patients with TIH exhibit evidence of altered regulation of NCC and AQP_2_.

The sodium-chloride cotransporter (i.e., NCC) is located in the renal cortex; therefore thiazides reduce maximal urinary diluting ability by increasing sodium excretion without affecting the renal medullary sodium gradient required for water reabsorption from the collecting duct.^7^ NCC exists in 3 isoforms.^8^ NCC_1_ and NCC_2_ vary by only 1 amino acid.^8^ NCC_3_, however is 9 amino acids longer; it constitutes approximately 40% of NCC is the human nephron. Regulation of NCC occurs principally by 2 mechanisms: regulation of apical membrane abundance (type 1 regulation) and regulation of transporter kinetics (type 2 regulation), the latter being controlled by phosphorylation of key N-terminal serine and threonine residues (Thr 60, Thr 55, and Ser 91).^9^, ^10^ Urinary concentration occurs principally through the vasopressin−AQP_2_ pathway in the collecting duct.^11^, ^12^, ^13^ Moreover, microperfusion studies suggest that thiazides may induce upregulation of AQP_2_, increasing the water permeability of the collecting duct.^14^, ^15^ Locally derived prostaglandin E_2_ (PGE_2_) is a key modulator of this pathway,^16^, ^17^ and its reuptake into collecting duct cells is determined by a PGE_2_-specific prostaglandin transporter (PGT). A recent study has suggested modest genetic associations with TIH, including aberrant PGE_2_ reuptake in the distal nephron that may increase AQP_2_-mediated water reabsorption in a subset of patients who carry a variant within *SLCO2A1*, the gene encoding PGT.^18^

Urinary extracellular vesicles (UEVs) are spherical, structured membrane vesicles that are formed by cells throughout the nephron.^19^ Because UEVs contain cell-derived membrane transporters and channels, their analysis is an attractive and noninvasive method to study renal tubular pathophysiology in patients.^20^, ^21^, ^22^

Because lithium is absorbed along with sodium throughout the nephron, it is possible to use measurement of lithium clearance to assess tubular sodium reabsorption. Renal lithium clearance likely reflects mostly proximal tubular sodium reabsorption, because this is where the majority of sodium is reabsorbed.^23^ Measuring endogenous lithium clearance (ELC) is preferable to exogenous lithium clearance following ingestion of lithium tablets, as it negates any potential effect which therapeutic doses of lithium may have on distal nephron physiology, including water trafficking,^24^ and is also more convenient for research volunteers.

To fulfill our aim of investigating whether patients with TIH exhibit evidence of altered regulation of NCC and AQP_2_, we report analysis of urinary extracellular vesicle NCC and AQP_2_ in TIH patients and controls. We also report assessment of renal tubular sodium reabsorption in TIH patients by measurement of ELC.

## Methods

### Clinical Recruitment

This study was conducted in line with the standards of ICH/Good Clinical Practise sections 8.2.8 in adherence to the Declaration of Helsinki and was approved by the UK National Research Ethics Committee (reference 11/EM/0233). Informed consent was given by all participants. Between April 2012 and August 2015, a daily search of the biochemistry database of all patients admitted to the department of internal medicine at Nottingham University Hospitals NHS Trust, UK, was undertaken to identify those patients with serum sodium <130 mM (cases). Patients were reviewed by the investigators to establish those whose hyponatremia was due to a thiazide diuretic (TIH). Normonatremic thiazide and nonthiazide controls (serum sodium 135−145 mM) were identified by primary care surgeries in Nottinghamshire and matched as closely as possible to the case patients for age, sex, comorbidities, and polypharmacy. Patients with TIH were also assessed in the outpatient clinic 2 months after thiazide cessation (termed normonatremic TIH case patients off thiazides). The patients described here were a subset of those previously reported^18^ and are composed of the small number of acutely hyponatremic TIH patients who were able to produce a mid-morning spot urine sample suitable for the current study.

### Urine Collection and Processing

Second-morning spot urine samples (30 ml) were collected in sterile containers from TIH patients and control groups (Supplementary Table S1). Protease inhibitor cocktail tablets (Roche Diagnostics) were added to urine samples immediately following collection and samples stored at −80°C. Fresh urine samples were processed the same day.

### Isolation of UEVs and Total Protein Estimation

Urinary nanovesicles, including UEVs, were isolated from both fresh and frozen urine by Ultrafiltration (Vivaspin 20 MWCO 100 kDa; Sartorius Stedim, Göttingen, Germany). Before processing of urine samples, nanomembrane concentrators were washed with phosphate-buffered saline solution to remove glycerol and other preservatives and then centrifuged at 2500 *g* for 5 minutes at room temperature. Frozen urine samples were thawed on ice, 1 ml dithiothreitol (100 mg/ml final concentration) was added, extensively vortexed and incubated for 30 minutes at 37 °C before UEV isolation. All samples were centrifuged (2500 *g* for 15 minutes at 4 ^o^C) for urinary cell removal. Cell pellet was suspended in lysis buffer (1.5% sodium dodecyl sulfate and 50 mM Tris-HCl with protease inhibitor, pH 6.8) and stored at −80 ^o^C. Further filtration of supernatant using 0.22-μm filters (Millipore, Watford, UK) was undertaken to remove debris. A 25-ml quantity of filtered supernatant was centrifuged at 15,000 *g* for 30 minutes at 4 °C. The urine supernatants were then added to Vivaspin (USA) nanomembrane concentrators and centrifuged at 4800 *g* for 1 hour at 4 °C. Concentrated UEVs were treated with 50 μl dithiothreitol, added directly into the filters, and incubated for 10 minutes at 37 °C. Isolated urinary extracellular vesicles were directly suspended in lysis buffer and stored at −80 °C. Total urine and flow-through fractions were also stored at −80 ^o^C and −20 ^o^C. Total soluble protein concentration was quantified with bicinchoninic acid assay (Pierce, Thermo Scientific, Paisley, UK).

### Electrophoresis and Western Immunoblotting

**Figure 1:**
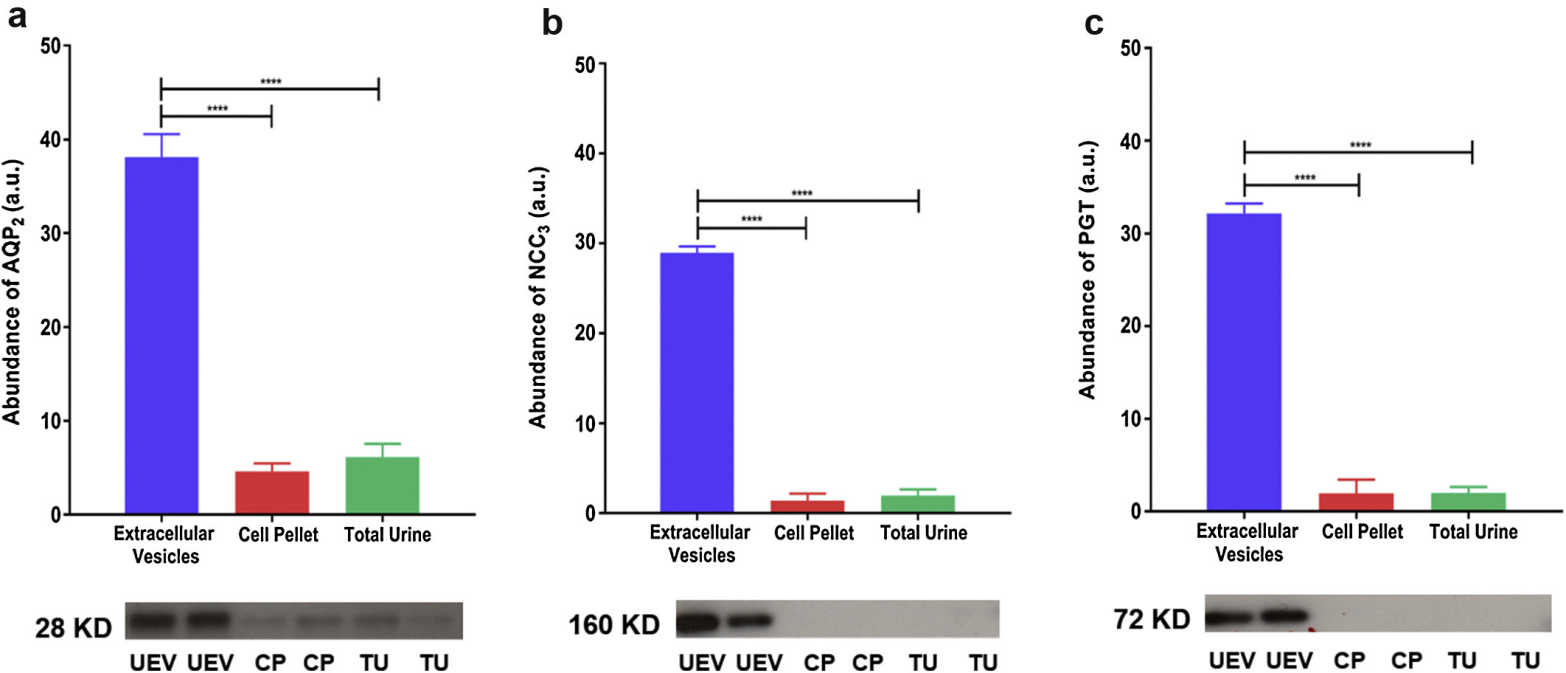
Western blot analysis of aquaporin-2 (AQP_2_), sodium-chloride cotransporter 3 (NCC_3_), and PGE_2_-specific prostaglandin transporter (PGT) in different urinary fractions demonstrates expression predominantly in urinary extracellular vesicles. An abundance of (a) AQP_2_, (b) NCC_3_, and (c) PGT is shown in urinary extracellular vesicles (UEVs), cell pellet (CP), and total urine (TU) fractions. Blots shown are representative of individual experiments; n = 8 in each group. Data are corrected for exosomal expression of apoptosis-linked gene 2-interacting protein X and are shown as mean ± SEM. ****P < 0.0001. a.u., arbitrary units.

**Figure 2:**
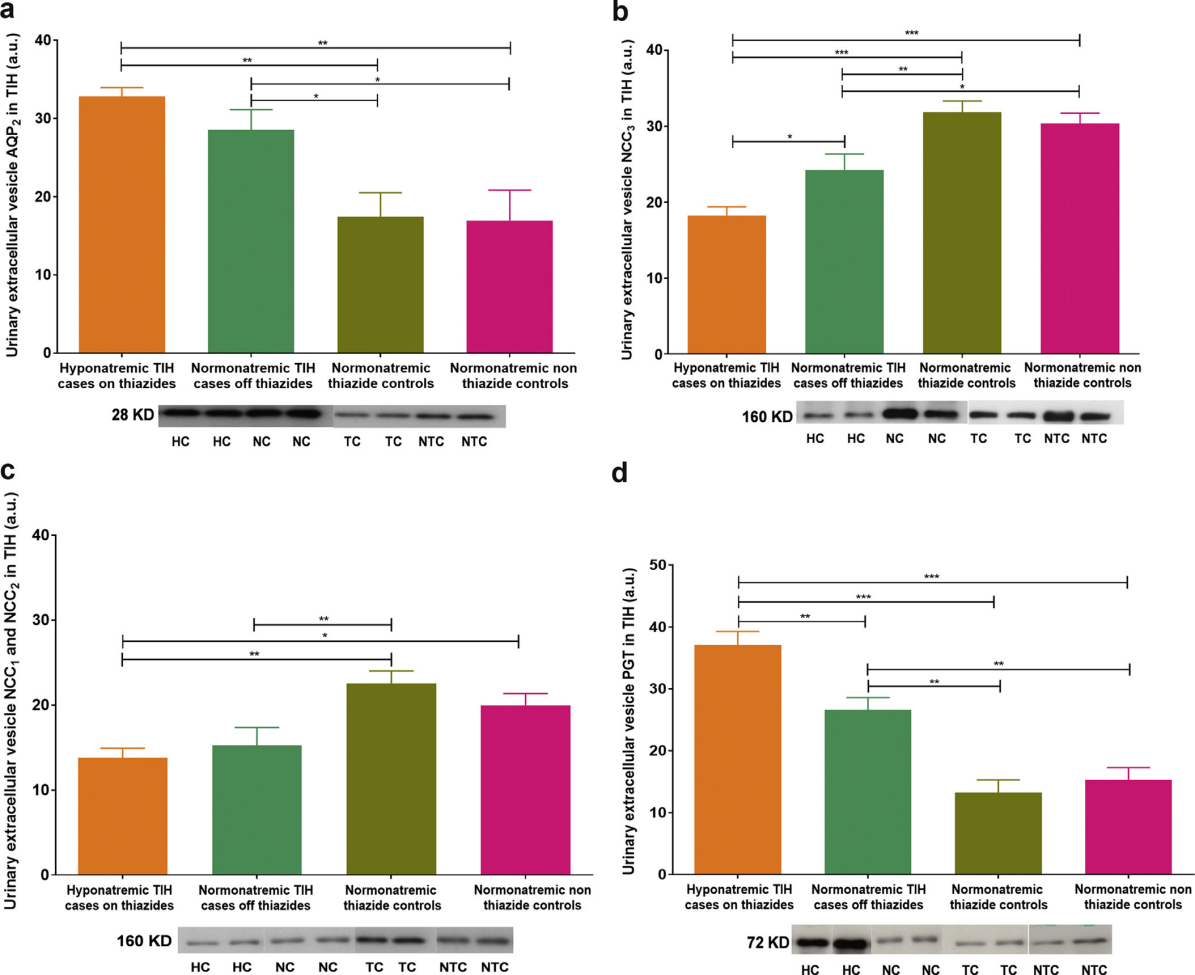
Western blot analysis of aquaporin-2 (AQP_2_), sodium-chloride cotransporter (NCC), and PGE_2_-specific prostaglandin transporter (PGT) in patients with thiazide-induced hyponatremia (TIH) and controls. Abundance of urinary extracellular vesicles (UEVs) (a) AQP_2,_ (b) NCC_3_, (c) NCC_1_ and NCC_2,_ and (d) PGT are shown. Blots shown are representative of individual experiments. Data are corrected for exosomal expression of apoptosis-linked gene 2-interacting protein X and are shown as mean ± SEM. *P < 0.05; **P < 0.01; ***P < 0.001. Hyponatremic TIH case patients on thiazides (HC; n = 8), normonatremic TIH case patients off thiazides (NC; n = 16), normonatremic nonthiazide controls (TC; n = 16), and normonatremic nonthiazide controls (NTC; n = 16) are represented. a.u., arbitrary units.

**Figure 3:**
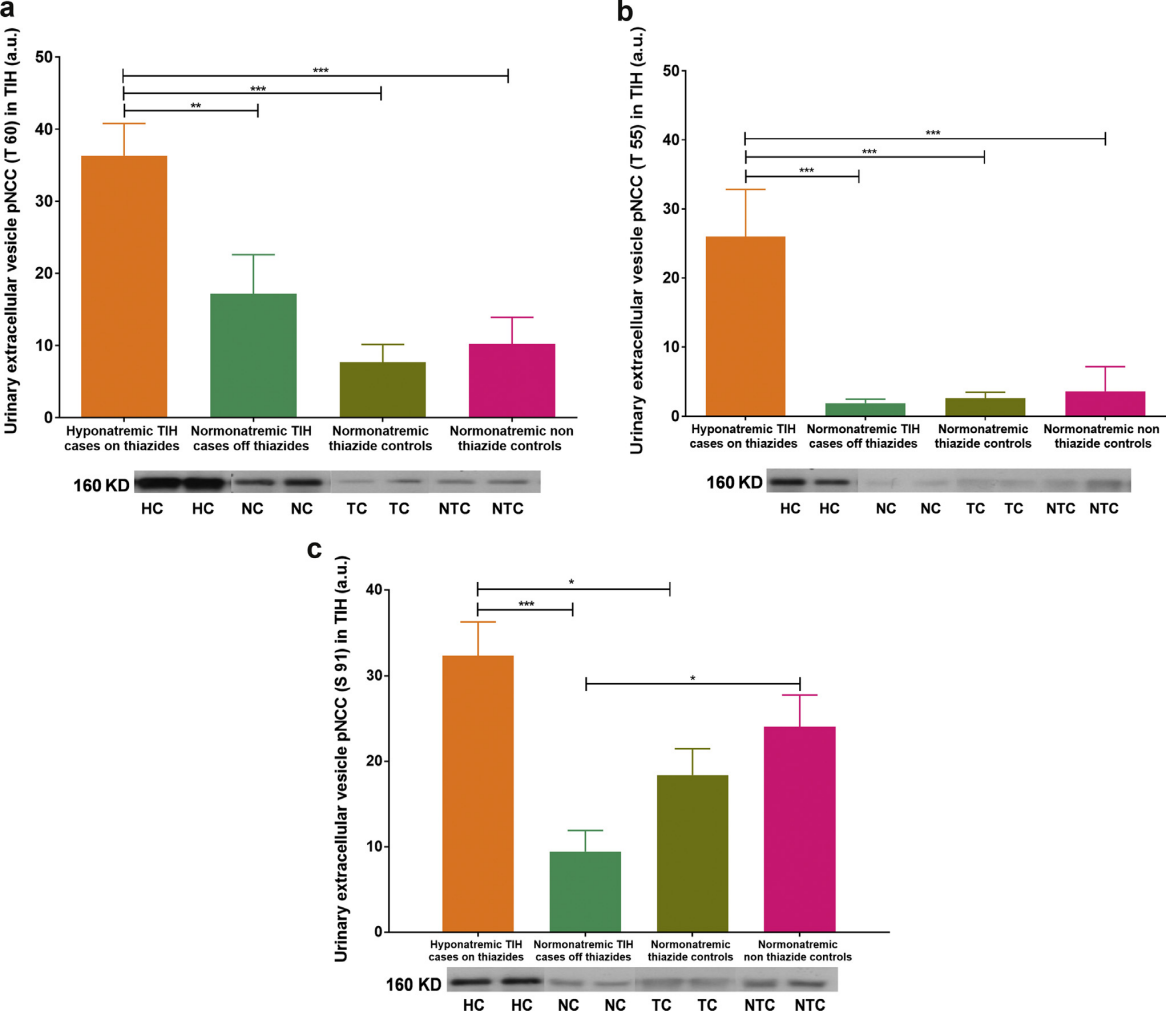
Western blot analysis of N-terminal sodium-chloride cotransporter (NCC) phosphorylation in thiazide-induced hyponatremia (TIH). Abundance of urinary NCC phosphorylation at (a) T60, (b) T55, and (c) S91 is shown in acute TIH case patients, convalescent TIH case patients, and controls on and off thiazide, respectively. Blots shown are representative of individual experiments. Data are corrected for exosomal expression of apoptosis-linked gene 2-interacting protein X and are shown as mean ± SEM. *P < 0.05, **P < 0.01, ***P < 0.001. Hyponatremic TIH case patients on thiazides (HC; n = 8), normonatremic TIH case patients off thiazides (NC; n = 16), normonatremic thiazide controls (TC; n = 16), and normonatremic nonthiazide controls (NTC; n = 16) are represented. a.u., arbitrary units.

A total of 25 μg soluble protein per sample was electrophoresed on 7.5 % (NCC and phospho-NCC), 10% (apoptosis-linked gene 2-interacting protein X [ALIX] and PGT) and 12.5% (AQP_2_) acrylamide gels. Sodium dodecyl sulfate−polyacrylamide gel electrophoresis was performed on polyvinylidene difluoride membrane (Bio-Rad, Watford, UK). Urinary extracellular vesicle AQP_2_ was detected by probing the blots with 1:500 diluted anti-AQP_2_ antibody (Sigma-Aldrich, Gillingham, UK) followed by anti-rabbit IgG horseradish peroxidase (HRP)−conjugated antibody (Sigma-Aldrich). Urinary extracellular vesicle NCC_3_ was probed with 1:500 diluted anti-NCC primary antibody (Millipore) followed by anti-rabbit IgG HRP−conjugated antibody (Sigma-Aldrich). Urinary extracellular vesicles NCC_1_ and NCC_2_ were probed with 1:500 diluted anti-NCC primary antibody (21st Century Biochemicals, Marlborough, MA) followed by anti-rabbit IgG HRP-conjugated antibody (Sigma-Aldrich). Urinary extracellular vesicle phospho-NCC (phosphorylated at T55, T60, and S91) was detected by probing blots with anti–phospho-NCC antibodies (University of Dundee, Dundee, UK), each at 1:500 (0.2–1.2 μg/ml) followed by anti-sheep IgG HRP-conjugated antibody (Sigma-Aldrich). To detect specific phosphorylated forms of NCC, the corresponding nonphosphorylated peptide was incubated with primary antibody (10 μg/ml). Urinary extracellular vesicle PGT was probed with 1:200 diluted anti-PGT primary antibody (Cayman, Cambridge, UK) followed by anti-rabbit IgG HRP-conjugated antibody (Sigma-Aldrich). The anti-AQP_2_ and NCC antibodies used target intracellular domains of AQP_2_ and NCC. Vesicles did not undergo prior permeabilization. Urinary cell pellet was used as a negative control for western blots. Urinary extracellular vesicle ALIX was detected by probing the blots with 1:1000 diluted anti-ALIX antibody (Abcam, Cambridge, UK) followed by anti-rabbit IgG HRP-conjugated antibody (Sigma-Aldrich). Western blot data were corrected for ALIX as indicated in the legends of Figures 1, 2, and 3. This method of correction by ALIX is as previously reported.^27^ The immunocomplex was detected by ECL chemiluminescence (GE Healthcare, Hatfield, UK) on X-Omat AR films (Kodak, Hemel Hempstead, UK). Normalization was carried using UEV ALIX, and bands were quantified using ImageJ (National Institutes of Health, Bethesda, MD) software. Density of each band was divided by the band from the same sample stained with Amido black. The differential expression of UEV proteins densitometry values among all different groups was evaluated by using 1-way analysis of variance followed by Tukey multiple comparison tests (GraphPad Prism V6.05, La Jolla, CA). A *P* value of <0.05 was considered significant.

### Nanoparticle Tracking Analysis

Nanoparticle tracking analysis (NTA) is a light-scatter microscopy method of tracking microparticles and nanoparticles on the basis of direct and real-time tracking of the particles’ Brownian movement, which results in a description of the particle size and concentration distribution in a given solution. Nanoparticle tracking analysis can be used to count and measure specific subgroups of nanoparticles using fluorescent antibodies against nanoparticle proteins, including extracellular vesicles derived from kidney cells in culture and in urine.^25^

Both AQP_2_ and NCC-expressing UEVs were analyzed using the NanoSight LM 10 instrument (NanoSight Ltd, Amesbury, UK). The analysis settings were optimized and kept constant between samples, and each video was analyzed to give the mean, mode, median, and estimated concentration for each particle size. All experiments were carried out at a 1:1000 dilution of unprocessed urine, yielding particle concentrations in the region of 1.3 × 10^8^ particles/ml in accordance with the manufacturer’s recommendations. For fluorescent NTA analysis, a 532-nm (green) laser diode excited the fluorescent-loaded ECVs with a long-pass filter (430 nm). For quantification of AQP_2_ (Millipore, Billerica, MA) and NCC (Chemicon USA, Temecula, CA), primary antibodies were fluorescent-labeled by conjugation to quantum dots. All samples were analyzed in triplicate to give 1 value per patient. Data for NCC and AQP_2_ were normalized by urinary creatinine.

### Endogenous Lithium Measurement

Serum and urine samples were prepared for analysis with a 1:25 dilution in 0.5% nitric acid (Romil, Cambridge, UK) containing 0.05% Triton-X 100 (Romil) and 40 ng/ml beryllium (Alfa Aesar, MA) as an internal standard. Measurements were performed on an Agilent 7900 ICP-MS (Agilent Technologies, La Jolla, CA) operated in no-gas mode with the radiofrequency generator power set to 950 W giving a “cold” plasma. Lithium was measured at m/z ratio of 7 and was normalized to the beryllium internal standard at m/z 9. Calibration solutions were prepared by diluting a 1000-μg/ml lithium standard (Alfa Aesar, Tewksbury, MA) in 2% nitric acid (Romil) to give concentrations of 0.0352, 0.141, 0.563, 2.25, 9, and 36 μmol/l. Seronorm trace elements urine quality control materials (Sero, Billingstad, Norway) and an in-house serum control were analyzed in each batch**.**

## Results

### Characterization and Validation of UEVs

Western blot analysis of AQP_2_, NCC_3_, and PGT was undertaken in healthy volunteers to establish (i) the principal urinary fraction(s) these transporters are excreted in UEVs, cell pellet, or total urine, and (ii) the optimal method of storage of urine samples for UEV analysis (fresh vs. frozen urine stored at either −20^o^C or −80 ^o^C).

The abundance of AQP_2_, NCC_3_, and PGT was much greater in UEVs than either cell pellet or total urine, confirming that UEVs are the most appropriate fraction of urine to study (Figure 1). The abundance of AQP_2,_ NCC_3_, and PGT in UEVs from freshly collected urine was not significantly different from that of urine that had been stored at −80 ^o^C with protease inhibitor (Supplementary Figure S1); however the AQP_2_ and NCC_3_ abundance was significantly less in the UEVs of urine stored at −20 °C compared to that from urine stored at −80 °C (Supplementary Figure S2).

### Expression of UEV AQP_2_, NCC, and PGT in TIH

The clinical characteristics of TIH patients and controls are shown in Supplementary Table 1. Urinary extracellular vesicle AQP_2_ was significantly more abundant in acutely hyponatremic TIH case patients on thiazides compared to normonatremic control patients on or off thiazides (Figure 2a). Even when TIH case patients had recovered to normonatremia, AQP_2_ expression was still greater than in controls (Figure 2a).

Urinary extracellular vesicle NCC_3_ and NCC_1_ and NCC_2_ were significantly less abundant in acutely hyponatremic TIH case patients on thiazides compared to convalescent normonatremic TIH case patients off thiazides and normonatremic control patients on or off thiazides (Figure 2b, c). Although NCC abundance increased in TIH case patients on recovery to normonatremia off thiazides, NCC expression was still 30% less than in normonatremic controls off thiazide (Figure 2b, c).

Extracellular vesicle prostaglandin transporter (PGT) was significantly more abundant in acutely hyponatremic TIH case patients on thiazides compared to normonatremic controls on thiazides. Despite reduction in PGT in TIH case patients following cessation of thiazide and recovery to normonatremia, PGT expression was still significantly greater than in normonatremic controls off thiazides (Figure 2d).

We also used a second method of UEV analysis, NTA, to analyze differences in NCC_3_ and AQP_2_ excretion between these patient groups. Nanoparticle tracking analysis has been used previously to quantify the abundance of UEVs containing AQP_2_^25^ and NCC_3_^26^ in unprocessed urine samples. Nanoparticle tracking analysis in our study showed that TIH patients had increased numbers of AQP_2_-positive UEVs in both acutely hyponatremic and convalescent normonatremic states compared to controls (Supplementary Figure S3). The numbers of NCC_3_-positive UEVs were not significantly different between the groups. Nanoparticle tracking analysis confirmed the changes in AQP_2_ expression in TIH patients seen by western blot analysis.

Assessment of NCC phosphorylation at 3 principal N-terminal locations known to accelerate transporter kinetics (T55, T60, and S91) was undertaken. Patients acutely hyponatremic on thiazides with TIH displayed significantly greater phosphorylation at T55, T60, and S91 than either convalescent normonatremic TIH case patients off thiazides or normonatremic controls on thiazides (Figure 3).

### Endogenous Lithium Clearance in TIH

**Figure 4:**
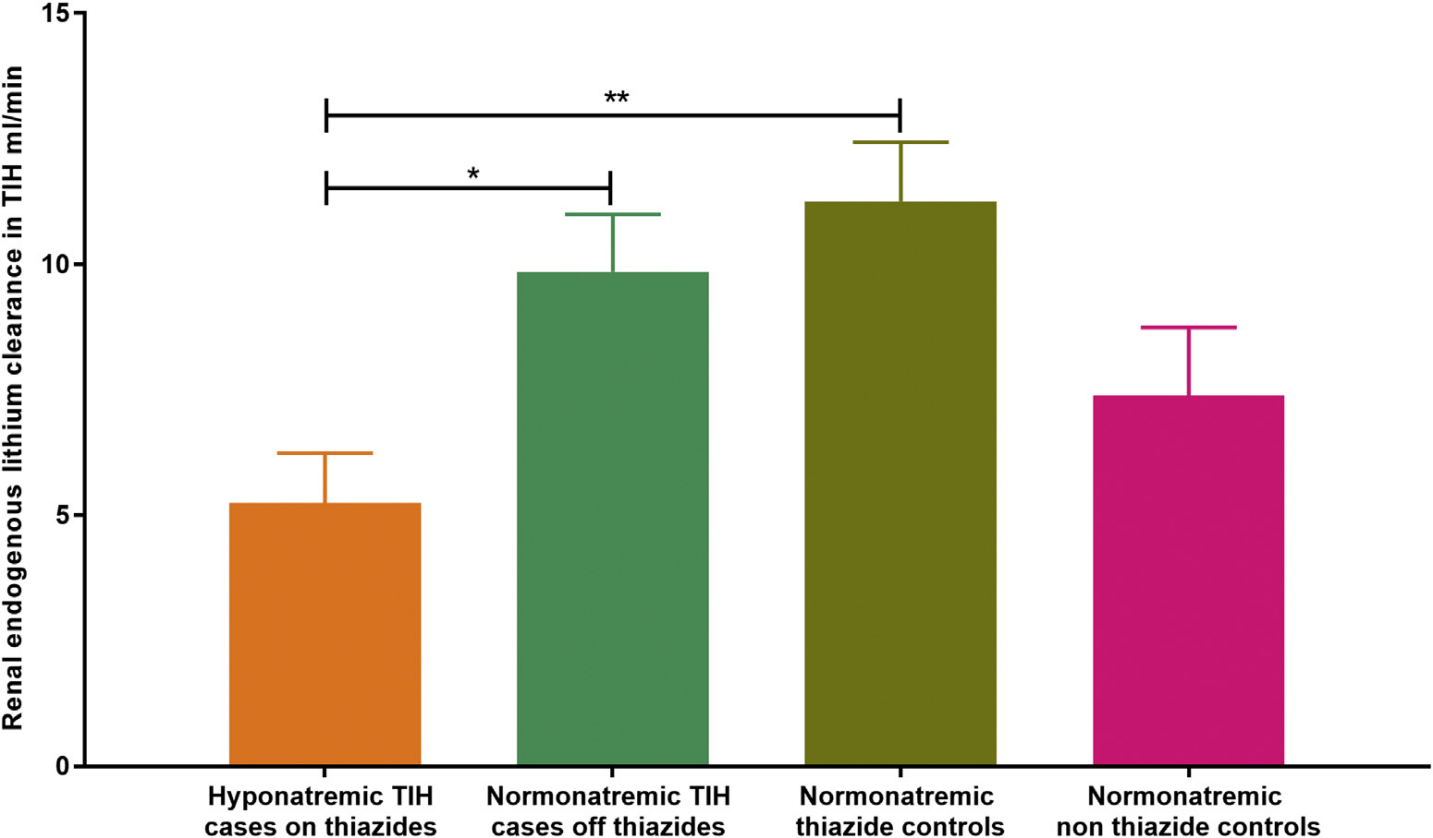
Renal endogenous lithium clearance (ELC) is reduced in acute thiazide-induced hyponatremia (TIH). The ELC was lower in hyponatremic TIH case patients on thiazides compared to either normonatremic TIH case patients off thiazides or normonatremic thiazide controls (n = 7 in each group). Data represented as mean ± SEM. *P < 0.05, **P < 0.01.

Renal endogenous lithium clearance (ELC) was lower in acutely hyponatremic TIH case patients on thiazides compared to convalescent TIH case patients on recovery to normonatremia following thiazide cessation (Figure 4). The ELC was also lower in acutely hyponatremic TIH case patients compared to normonatremic controls on thiazide (Figure 4).

## Discussion

Here we report a noninvasive method of obtaining, preserving, and analyzing urinary extracellular vesicles from volunteer patients with thiazide-induced hyponatremia who were admitted to an acute internal medicine department. In addition, analysis of distal nephron sodium, water, and prostaglandin transporters contained within the extracellular vesicles suggests a possible mechanism that may underlie the pathophysiology of TIH. These data add to what has previously been reported^18^ by demonstrating that patients with acute TIH display reduced total NCC and increased phospho-NCC and AQP_2_ in their UEVs relative to appropriate control groups.

We have shown that the extracellular vesicles fraction is the most appropriate component of human urine with which to study the expression of NCC, AQP_2_, and PGT. Furthermore, we demonstrate that it is not necessary to analyze only fresh urine samples, and that samples frozen with protease inhibitor are equally suitable for use. This is important, because if analysis were limited to fresh samples, the time-consuming sample processing and immediate exosome isolation would make the study of acute medical conditions such as TIH very difficult. Because acquisition of a spot urine sample, addition of a protease inhibitor tablet, and storage in a freezer can be performed by the majority of clinical and research staff with very limited training required, such sample acquisition from acute medical patients becomes a much more feasible proposition.

*A priori* TIH must result from excessive saliuresis and/or water reabsorption. As thiazide diuretics have not been observed to produce saliuresis with urinary sodium concentration greater than those of plasma, excess water reabsorption (or ingestion) is likely to be a fundamental part of TIH pathophysiology. Reduced renal expression of NCC and increased expression of AQP_2_ in acute TIH would support the hypothesis that the pathophysiology of TIH results from a combination of both of these processes. We have successfully identified both NCC_1_ and NCC_2_, NCC_3_ and determined the phosphorylation status of 3 key N-terminal phosphorylation sites known to regulate NCC activity from the urinary extracellular vesicles. In addition, reduced endogenous lithium clearance (ELC) in acute TIH supports increased proximal sodium reabsorption reflecting augmented distal nephron sodium loss, and so is complementary to the UEV data.

The increased N-terminal phosphorylation state of NCC in acute TIH suggests some degree of physiological compensation to excessive distal nephron sodium loss, by attempting to maximally activate what NCC exists in the distal convoluted tubule. Why type 1 regulation of NCC (apical membrane expression) should be impaired in TIH but type 2 regulation (transporter activation by N-terminal phosphorylation) should remain intact is unclear. It is notable that thiazides inhibit NCC by binding to the cotransporter from the luminal membrane, and 1 hypothesis may be that the physical binding of thiazides to NCC induces a conformational change that targets the cotransporter for endocytosis and degradation in patients susceptible to TIH. Alternatively, saliuresis and resulting kaliuresis and trend to hypokalemia seen in acute TIH patients may drive phosphorylation and activation of remaining NCC.^27^

Thiazides reduce the ability of the late diluting segment (distal convoluted tubule) to generate solute-free water directly, and also act by reducing effective vascular volume and thus solute delivery from the end-proximal tubule.^28^ The increased proximal renal sodium reabsorption observed would support a reduction in the amount of fluid available to reach the diluting segments of the nephron, which is then further reduced by the role of thiazides and the observed reduction in NCC expression acting to reduce dilution. Study of NHE3 expression in the proximal tubule may therefore be informative for future studies.

Measuring differences in the UEV profile of acutely hyponatremic patients may also have clinical utility as an aid to TIH diagnosis where other potential contributors to hyponatremia exist. The use of UEV analysis as a diagnostic tool would require further studies to identify whether acute differences in UEV profiling exist in other causes of hyponatremia and whether profiling of a panel of UEV markers could reliably differentiate 1 cause of hyponatremia from another.

We chose also to study PGT because it is constitutively expressed in the collecting duct, where it mediates PGE_2_ uptake, 1 of the most important physiological regulators of distal nephron water reabsorption.^28^ The protein-altering variant in *SLCO2A1* (p.A396T), the gene that encodes PGT, was also recently reported to show modest association with TIH.^18^ Here we show that PGT is significantly expressed in UEVs and that PGT expression is substantially elevated in acute TIH. This provides further support that PGT may be involved in the molecular pathways causing inappropriate water reabsorption in TIH.

That differences in the extracellular vesicle profile of convalescent normonatremic TIH cases exist 2 months after thiazides have been discontinued suggests that patients who are predisposed to TIH may exhibit these urinary extracellular vesicle characteristics before they are exposed to thiazides. Although a prospective study would be required to confirm this observation, it raises the possibility that pre-prescription profiling of the relative abundance of NCC to AQP_2_ could potentially identify patients at higher risk for TIH. These individuals could then be offered alternative antihypertensive therapy, for example, medications from the angiotensin-converting enzyme inhibitor or calcium channel antagonist group; or, if a diuretic is required, an alternative loop or potassium-sparing type could be selected instead of a thiazide.

In summary, we show here that TIH patients display reduced NCC expression and increased AQP_2_ expression in their UEVs, which would be expected to produce excessive saliuresis and water resorption. Reduced ELC also suggests compensatory increased proximal sodium reabsorption in TIH. That differences in the renal transporters responsible for these processes can be detected in urinary extracellular vesicles both acutely and following recovery from TIH raises the possibility that such techniques may have utility in prospectively identifying those individuals at high risk for TIH and in aiding the acute diagnosis of TIH in cases that are confounded by comorbidity and/or polypharmacy.

## Disclosure

All the authors declared no competing interests.

## Supplementary Material

Table S1 Clinical characteristics of TIH patients and controls.

Figure S1 Western blotting of urinary extracellular vesicle demonstrates that abundance of (A) AQP2, (B) NCC3, and (C) PGT is not significantly different in fresh (F) versus frozen (FR) urine.

Figure S2 Western blotting of urinary extracellular vesicle demonstrates that abundance of (A) AQP2 and (B) NCC is significantly different in urine stored at −80°C versus −20°C.

Figure S3 Nanoparticle tracking analysis.

Figure S4 Western blotting analysis of urinary extracellular vesicle (A) AQP2 (B) NCC, and (C) PGT in TIH patients and controls.

Figure S5 Western blotting analysis of urinary extracellular vesicle phospho-NCC (A) T60, (B) T55, and (C) S91 in TIH patients and controls.
