# Supplementary material for: Urinary Extracellular Vesicle Protein Profiling and Endogenous Lithium Clearance Support Excessive Renal Sodium Wasting and Water Reabsorption in Thiazide-Induced Hyponatremia
Source: Kidney Int Rep. 2018 Sep 22;4(1):139–47. doi: 10.1016/j.ekir.2018.09.011 (PMC6308385; doi:10.1016/j.ekir.2018.09.011)
Supplement: Table S1 — Clinical characteristics of TIH patients and controls. [file mmc1.docx]

**Supplementary material**

| Clinical Characteristic | TIH cases | | Controls | |
| --- | --- | --- | --- | --- |
|  | Hyponatremic TIH cases on thiazides | Normonatremic TIH cases off thiazides | Normonatremic thiazide controls | Normonatremic non-thiazide controls |
| Number | 8 | 16 | 16 | 16 |
| Age years(±SD) | 81 (+/-9) | 80 (+/-8) | 76 (+/-7) | 74 (+/-7) |
| Female (%) | 63 | 63 | 63 | 63 |
| ACEi or ARB (%) | 50 | 56 | 50 | 56 |
| β-blocker (%) | 25 | 31 | 37.5 | 31 |
| CCB (%) | 25 | 62 | 50 | 44 |
| loop diuretic (%) | 12.5 | 6 | 0 | 6 |
| K^+^ sparing diuretic (%) | 0 | 6 | 0 | 6 |
| SSRI (%) | 0 | 12.5 | 0 | 6 |
| Serum sodium (mM) (±SD) | 120 (+/-7.3) | 137 (+/-3.1) | 138 (+/-3.7) | 139 (+/-2.2) |
| Serum potassium (mM) (±SD) | 3.6 (+/-0.4) | 4.2 (+/-0.6) | 4.0(+/- 0.6) | 3.8 (+/-0.5) |
| Serum creatinine (µM) (±SD) | 87 (+/-22) | 63 (+/-16) | 84 (+/-10) | 79 (+/-15) |
| Serum osmolarity  mosmol/Kg (±SD) | 256 (+/-12) | 286(+/-9) | 297 (+/-8) | 291 (+/-7) |
| Urinary sodium (mM) (±SD) | 29 (+/-15) | 65(+/-12) | 64 (+/-13) | 62 (+/-23) |
| Urinary potassium (mM) (±SD) | 18 (+/-8) | 52(+/-16) | 45 (+/-14) | 40 (+/-11) |
| Urinary creatinine (mM) (±SD) | 5 (+/-2) | 6(+/-3) | 9 (+/-3) | 5.8 (+/-2) |
| Urinary osmolarity  mosmol/Kg (±SD) | 345(+/-89) | 323(+/-82) | 398 (+/-94) | 413 (+/-81) |
| **Comorbidities** |  |  |  |  |
| Treated hypothyroidism (%) | 0 | 0 | 6 | 6 |
| Diabetes Mellitus, glucose <13.9mM (%) | 0 | 12.5 | 12.5 | 6 |
| Mild LV impairment (%) | 12.5 | 12.5 | 0 | 0 |
| **Thiazide type** |  |  |  |  |
| BFZ (%) | 62.5 |  | 75 |  |
| Chlortalidone (%) | 0 |  | 12.5 |  |
| Indapamide (%) | 37.5 |  | 12.5 |  |
| HCTZ (%) | 0 |  | 6 |  |
|  |  |  |  |  |

**Supplementary table 1. Clinical characteristics of TIH patients and controls.** All patients were Caucasian. Standard Deviation (SD), Angiotensin Converting Enzyme Inhibitor (ACEi), Angiotensin II Receptor Blocker (ARB), β-adrenoceptor blocker (β-blocker), Calcium Channel Blocker (CCB), potassium sparing diuretic (K^+^ sparing diuretic), Selective Serotonin Reuptake Inhibitor (SSRI), Non-Steroidal Anti-Inflammatory Drug (NSAID), Bendroflumethiazide (BFZ), HydroChloroThiazide (HCTZ). Treated hypothyroidism (no clinical features of hypothyroidism and TSH within normal range), diabetes mellitus (plasma glucose<13.9mM), mild Left Ventricular (LV) impairment (no clinical features of heart failure and ECHO mild LV impairment).
