# Supplementary material for: Urinary Extracellular Vesicle Protein Profiling and Endogenous Lithium Clearance Support Excessive Renal Sodium Wasting and Water Reabsorption in Thiazide-Induced Hyponatremia
Source: Kidney Int Rep. 2018 Sep 22;4(1):139–47. doi: 10.1016/j.ekir.2018.09.011 (PMC6308385; doi:10.1016/j.ekir.2018.09.011)
Supplement: Figure S1 — Western blotting of urinary extracellular vesicle demonstrates that abundance of (A) AQP2, (B) NCC3, and (C) PGT is not significantly different in fresh (F) versus frozen (FR) urine. [file mmc2.docx]

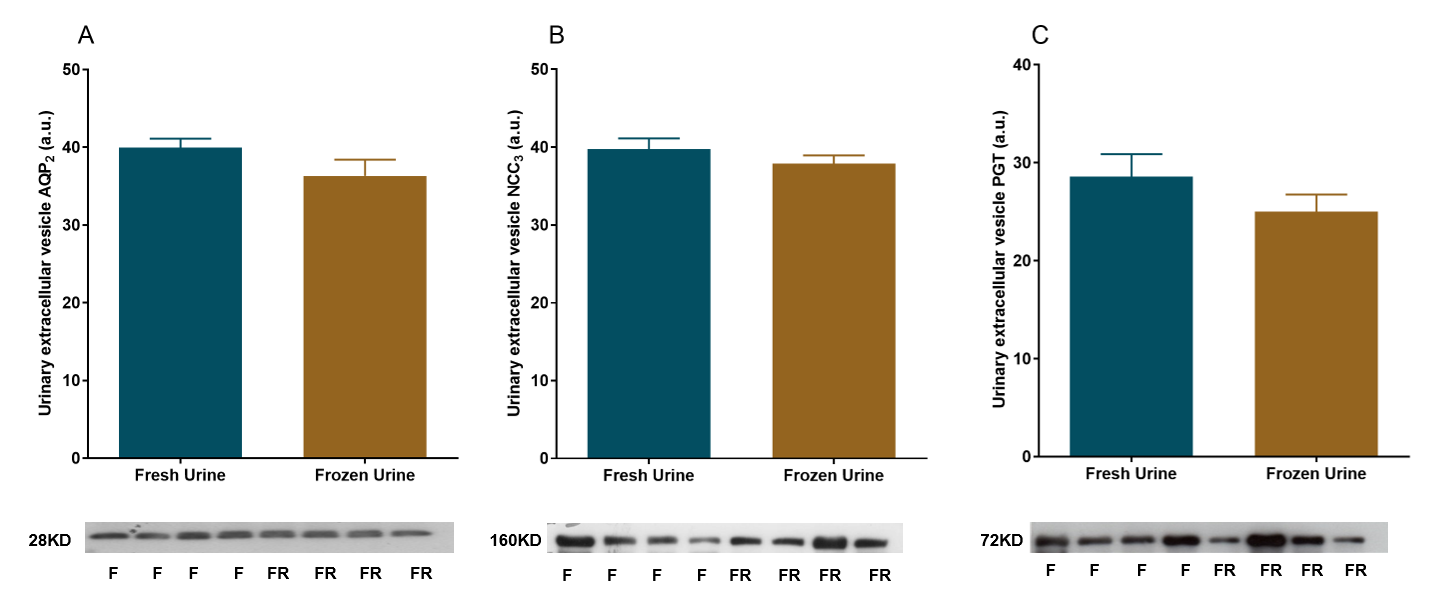
**Supplementary Figure 1: Western blotting of urinary extracellular vesicle demonstrates that abundance of (A) AQP_2,_ (B) NCC_3_ and (C) PGT is not significantly different in fresh (F) vs. frozen (FR) urine.** Samples were from healthy volunteers. Frozen urine samples were stored at -80^o^C. Blots shown are representative of individual experiments. N=8 in each group. Data are corrected for ALIX and are shown as mean ± SEM.


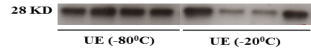

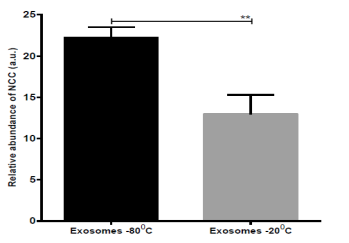

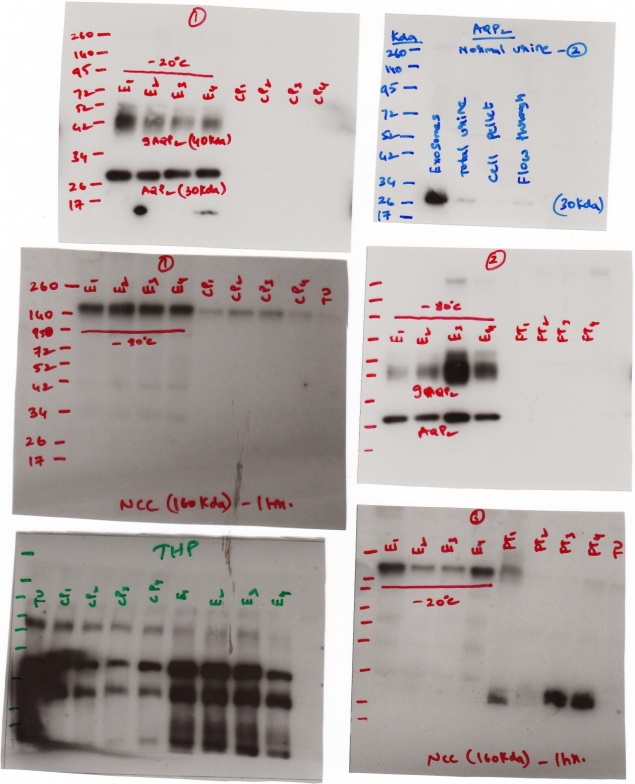


**UE (-20^0^C)**


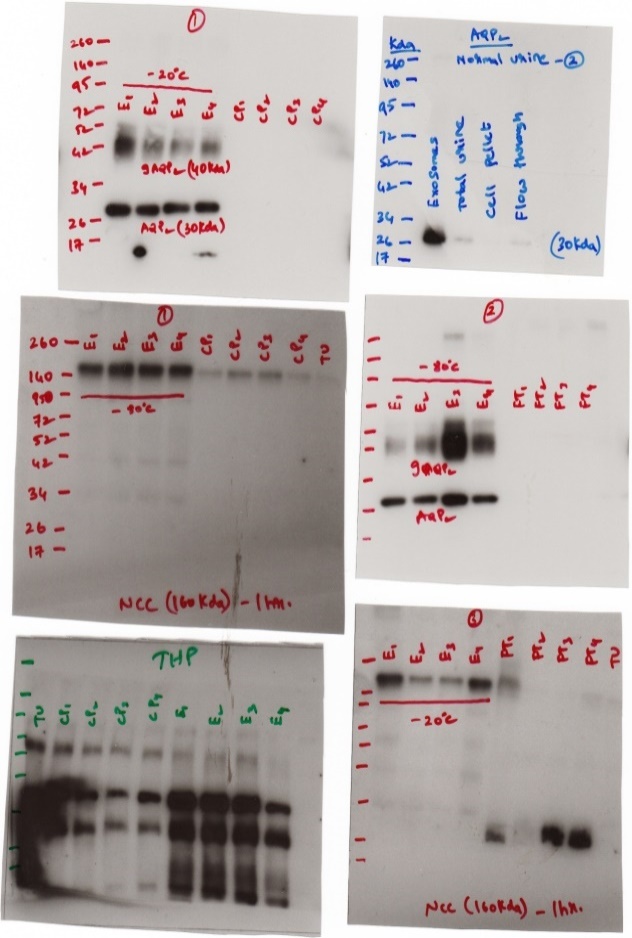


**UE (-80^0^C)**

**160KD**

**A**

**B**


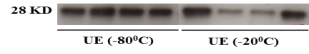


**28KD**

**UE (-20^0^C)**

**UE (-80^0^C)**
