# Supplementary material for: Urinary Extracellular Vesicle Protein Profiling and Endogenous Lithium Clearance Support Excessive Renal Sodium Wasting and Water Reabsorption in Thiazide-Induced Hyponatremia
Source: Kidney Int Rep. 2018 Sep 22;4(1):139–47. doi: 10.1016/j.ekir.2018.09.011 (PMC6308385; doi:10.1016/j.ekir.2018.09.011)
Supplement: Figure S2 — Western blotting of urinary extracellular vesicle demonstrates that abundance of (A) AQP2 and (B) NCC is significantly different in urine stored at −80°C versus −20°C. [file mmc3.docx]

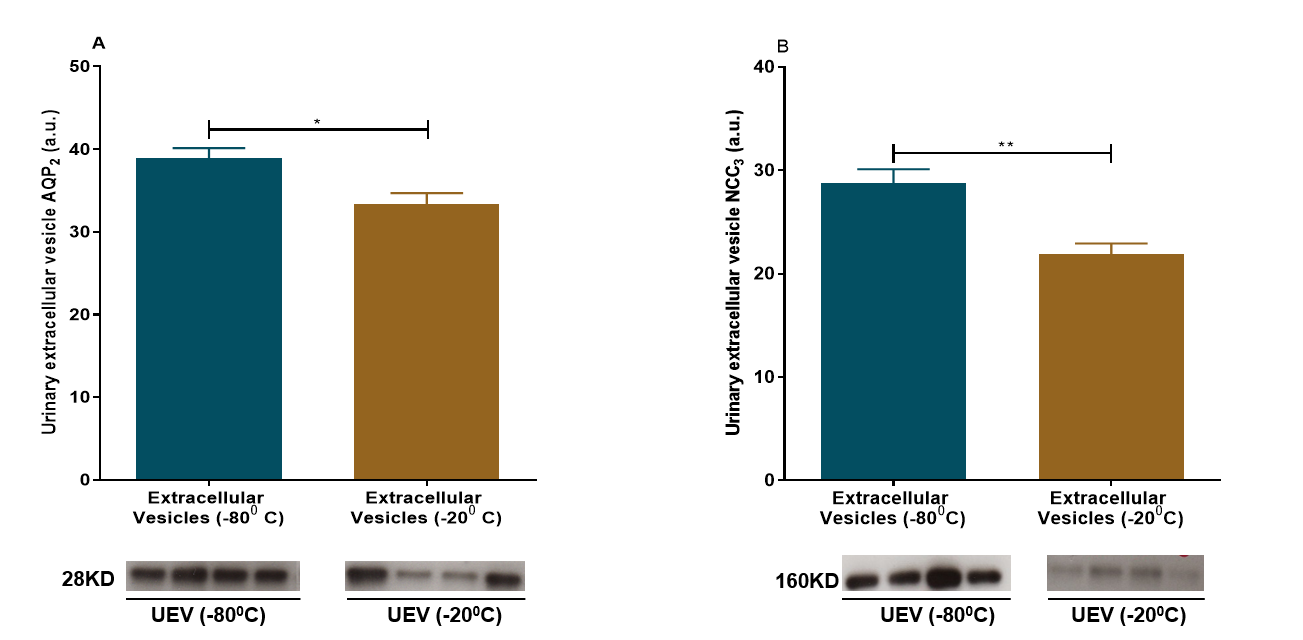


**Supplementary** **Figure 2:** **Western blotting of urinary extracellular vesicle demonstrates that abundance of (A) AQP_2_ and (B) NCC is significantly different in urine stored at -80^0^C vs. -20^0^C.** Samples were from healthy volunteers. Frozen urine samples were stored at -80^o^C. Blots shown are representative of individual experiments. N=8 in each group. Data are corrected for ALIX and are shown as mean ± SEM. * =p<0.05, **=p<0.01
