# Supplementary material for: Urinary Extracellular Vesicle Protein Profiling and Endogenous Lithium Clearance Support Excessive Renal Sodium Wasting and Water Reabsorption in Thiazide-Induced Hyponatremia
Source: Kidney Int Rep. 2018 Sep 22;4(1):139–47. doi: 10.1016/j.ekir.2018.09.011 (PMC6308385; doi:10.1016/j.ekir.2018.09.011)
Supplement: Figure S3 — Nanoparticle tracking analysis. [file mmc4.docx]

**
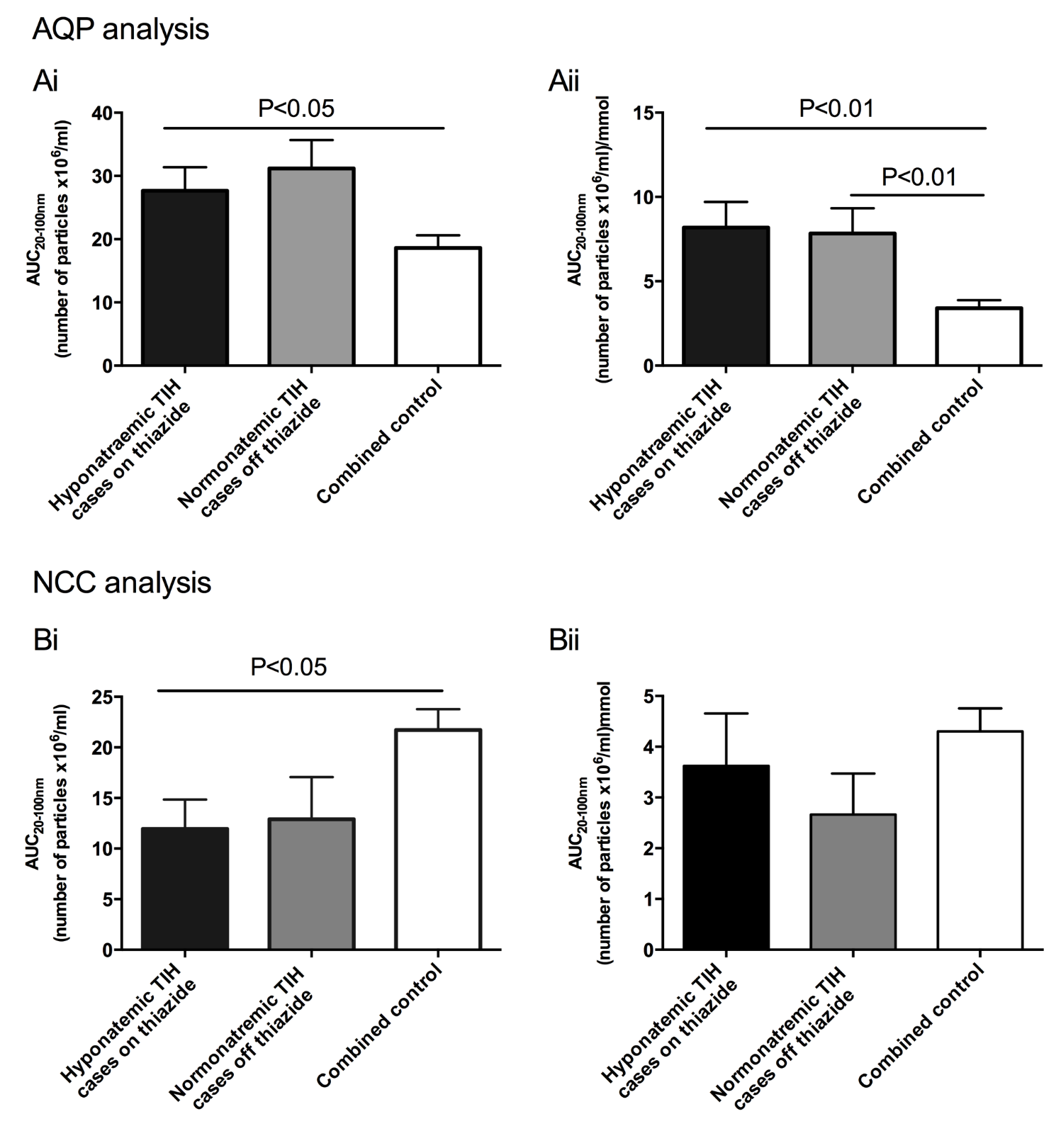
**

**Supplementary Figure 3: Nanoparticle Tracking Analysis was used to count the number of urinary exosomes expressing A) AQP2 and B) NCC in TIH patients on thiazide (Black bar; n=6) and TIH patients off thiazide (Grey bar; n=6).** Data are shown (i) without and (ii) with normalisation to urinary creatinine. Combined controls; (white bar; n=12) represent normonatremic patients on and off thiazide). Data are mean±SE. One-way ANOVA indicated a significant difference between groups for both AQP2-containing exosomes (normalised to urinary creatinine, F=7.884 P= 0.0028, and for uncorrected data F= 4.962, and P= 0.0172) and NCC-containing exosomes (normalised to urinary creatinine F= 1.450, P=0.2572, and for uncorrected data F=4.114, P=0.0311); Bonferroni post-hoc analysis for defined comparisons is shown (denoted by the P values in the figures).
