# Supplementary material for: Urinary Extracellular Vesicle Protein Profiling and Endogenous Lithium Clearance Support Excessive Renal Sodium Wasting and Water Reabsorption in Thiazide-Induced Hyponatremia
Source: Kidney Int Rep. 2018 Sep 22;4(1):139–47. doi: 10.1016/j.ekir.2018.09.011 (PMC6308385; doi:10.1016/j.ekir.2018.09.011)
Supplement: Figure S5 — Western blotting analysis of urinary extracellular vesicle phospho-NCC (A) T60, (B) T55, and (C) S91 in TIH patients and controls. [file mmc6.docx]

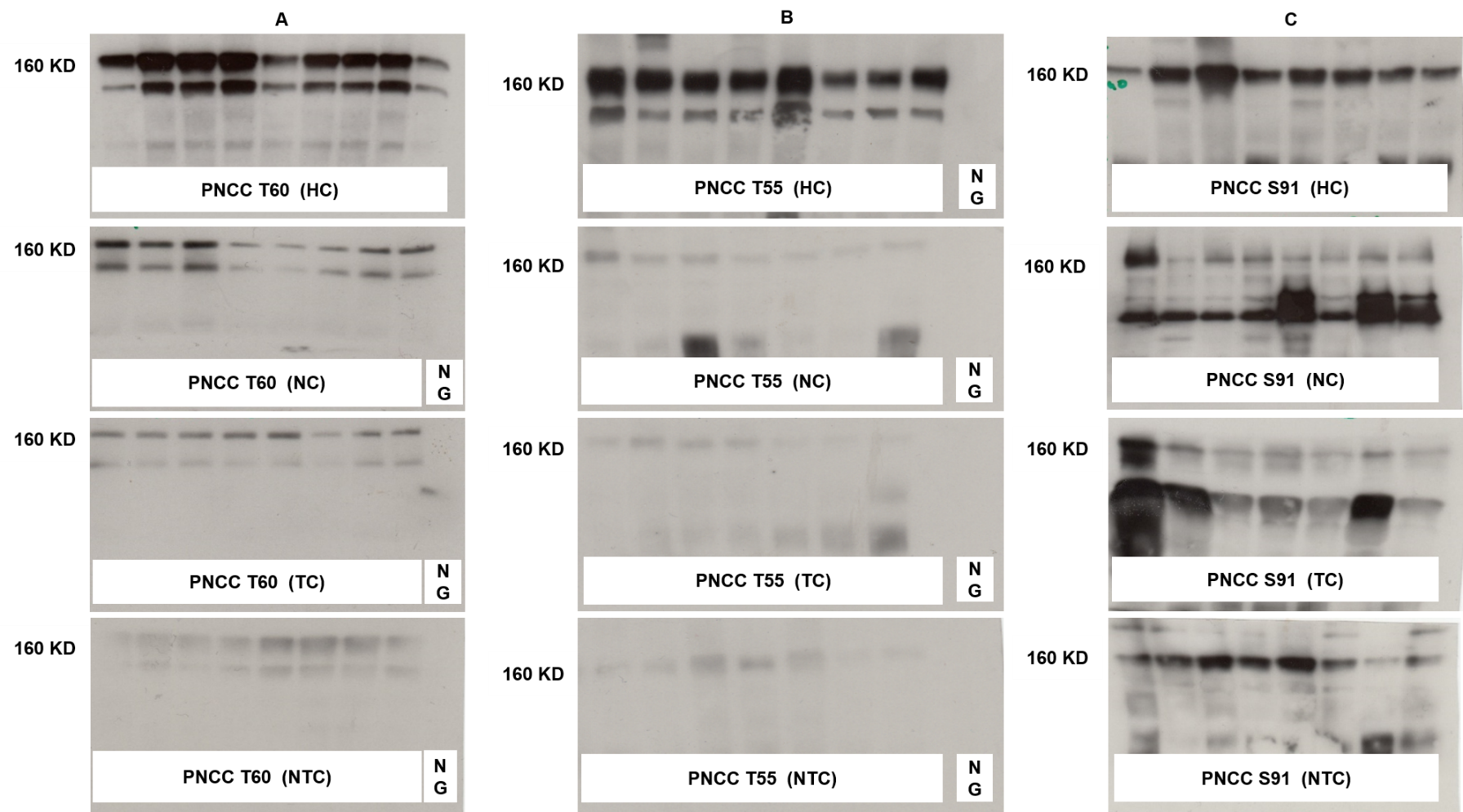


**Supplementary** **Figure 5:** **Western blotting analysis of urinary extracellular vesicle pNCC (A) T60 (B) T55 and (C) S91 in TIH patients and controls**. Hyponatremic TIH cases on thiazides (HC), normonatremic TIH cases off thiazides (NC), normonatremic thiazide controls (TC) and normonatremic non-thiazide controls (NTC) and NeGative control (NG).
